# A Review of Oxidative Stress and Urinary Dysfunction Caused by Bladder Outlet Obstruction and Treatments Using Antioxidants

**DOI:** 10.3390/antiox8050132

**Published:** 2019-05-15

**Authors:** Yasuyoshi Miyata, Tomohiro Matsuo, Kensuke Mitsunari, Akihiro Asai, Kojiro Ohba, Hideki Sakai

**Affiliations:** Department of Urology, Nagasaki University Graduate School of Biomedical Sciences, Nagasaki 852-8501, Japan; tomozo1228@hotmail.com (T.M.); ken.mitsunari@gmail.com (K.M.); rsjfk462@yahoo.co.jp (A.A.); ohba-k@nagasaki-u.ac.jp (K.O.); hsakai@nagasaki-u.ac.jp (H.S.)

**Keywords:** oxidative stress, bladder dysfunction, bladder outlet obstruction, antioxidants

## Abstract

Urinary dysfunction is a common pathological condition that can significantly decrease the quality of life. Bladder outlet obstruction (BOO) is a major cause of urinary dysfunction, and various lower urinary tract diseases including benign prostatic hyperplasia and urethral stricture disease cause BOO. According to the results of a variety of animal experiments on partial BOO (PBOO), there is a general agreement that ischemic conditions and repeated ischemia/reperfusion of the bladder are closely associated with BOO-induced bladder damage, and that increased oxidative stress by ischemia/reperfusion plays a crucial role in the pathological mechanisms underlying urinary dysfunction. Changes in biomarkers of oxidative stress in PBOO animal models support this association between oxidative stress and urinary dysfunction. Oxidative stress is defined as an imbalance between the production of pro-oxidants, such as free radicals and reactive species, and their elimination through protective mechanisms of antioxidants. Therefore, organizing the knowledge on the state of oxidative stress, changes in biomarkers, and biological roles of antioxidants in systemic and bladder tissues is essential to understand the detailed pathological characteristics of the urinary dysfunction caused by PBOO. Furthermore, information on drugs and supplements that have antioxidant effects is important for defining treatment strategies for urinary dysfunction with PBOO. In this review, we paid special attention to the following three issues; (1) changes in oxidative stress, including its biomarkers, (2) antioxidant status, and (3) previous reports on treatment strategies involving agents with antioxidative activity for urinary dysfunction caused by BOO. In particular, we provide systematic information on the detailed mechanisms underlying the antioxidative effects of agents used to treat PBOO. In addition, we show present research issues and research limitations, as well as suggest possible future antioxidant treatment strategies for patients with PBOO.

## 1. Introduction

Oxidative stress plays crucial roles in the development of many chronic diseases, including neurodegenerative diseases, cardiovascular disease, and malignancies [1,2,3,4]. It also plays an important role not only in biochemistry and cell biology but also in the nutritional sciences, environmental medicine, and molecular knowledge-based redox medicine, and therefore research on this topic is relevant for the maintenance of a heathy condition, for use of medicines, and for improved understanding of various diseases [5,6]. In addition, oxidative stress is closely associated with pathological mechanisms and symptoms of urinary bladder dysfunction [7,8,9,10,11,12,13]. Therefore, detailed and extensive knowledge about the pathological significance of oxidative stress in the bladder is important to understand the cause of diseases and potential treatment strategies for patients with urinary dysfunctions. 

Oxidative stress related to increasing intracellular levels of reactive oxygen species (ROS), which is a heterogeneous group of highly reactive ions and molecules derived from molecular oxygen (O_2_), including superoxide anions, hydroxyl radicals, hydrogen peroxide, hypochlorous acid, and singlet oxygen [14]. In addition, reactive nitrogen species (RNS) are well-known to play crucial roles in certain physiological and pathological conditions, including the cardiovascular system and in malignancies [15,16]. Although ROS/RNS are believed to be toxic and cause significant damage in various target organs in the traditional view, subsequent studies have shown that they also can play beneficial roles in physiological processes [15,17]. The degree of ROS/RNS production may underlie this paradox [18,19,20]. In short, ROS/RNS contribute to adequate controlled proliferation, apoptosis, differentiation, migration, and metabolism in physiological conditions; however, excessive production of ROS/RNS can cause oxidative stress, which may damage cellular lipids, proteins, cell membrane, and DNA, thereby changing the structure and function of target tissues [18,19,20,21]. In fact, low and moderate concentrations of ROS/RNS are useful for ordered cellular signaling and mitogenic responses, whereas additional production leads to various pathological conditions including cancers, diabetes, and cardiovascular diseases [3,22]. The balance of ROS/RNS levels is tightly controlled by regulation of the production and elimination of ROS/RNS endogenous antioxidants that act as scavengers under physiological conditions. In other words, ROS/RNS are major players in the etiology of various diseases caused by oxidative stress, and some substances with antioxidative effects may improve such pathological conditions.

Partial bladder outlet obstruction (PBOO) is a common pathological condition in older people, which results from various lower urinary tract diseases such as benign prostatic hyperplasia (BPH) and urethral stricture. Importantly, PBOO is a cause of voiding dysfunction, detrusor overactivity, vesicoureteral reflux, urinary tract infection, and overactive bladder, because it usually leads to significant structural and functional changes in the bladder [22,23]. Importantly, urinary dysfunction caused by PBOO often decreases the quality of life by inducing pollakiuria, urgency, and residual urine, thereby subsequent insomnia, lower abdominal discomfort, and incontinence. At present, there is a general agreement that oxidative stress plays important roles for pathological mechanisms of PBOO. 

In this review, we introduce the pathological roles of oxidative stress including ROS, changes in biomarkers by PBOO, and the state of antioxidants during urinary dysfunction. In vivo data on treatment strategies based on agents with antioxidative effects are also discussed. In urinary dysfunction caused by PBOO, pathological activities and regulative mechanisms of oxidative stress are extremely complex and much is unknown. In fact, useful treatments that rely on antioxidants have not yet been developed. However, we believe that a summary of the pathological significance of oxidative stress and results of preliminary trials in animal models is important to guide the future direction of treatment strategies in patients with PBOO.

## 2. Oxidative Stress Including Reactive Oxygen Species in Bladder Outlet Obstruction

For almost all organs, including the urinary bladder, an adequate blood supply is essential to obtaining oxygen and nutrition for functioning properly. Ischemic conditions decrease organ function. In PBOO, a reduction of blood flow and chronic ischemia in the bladder has been demonstrated in various animal models [9], and similar findings have been confirmed in human bladder tissues [24]. Such an ischemic status in the bladder is followed by an increase in bladder flow after micturition. In addition, such ischemia that accompanies hypoxia and ischemia/reperfusion during a micturition cycle is repeated in the bladder. At present, evidence suggests that cyclic ischemia/reperfusion may lead to bladder outlet obstruction (BOO)-induced bladder dysfunction. And a resulting increase in oxidative stress is associated with such pathological mechanisms underlying bladder damage [7,9,10,11,12]. PBOO has been shown to increase systemic oxidative stress in animal experiments and is reported to affect bladder function via bladder ischemia- and ischemia/reperfusion-induced oxidative stress in various examinations including animal experiments [8,12,13,25]. 

**Figure 1:**
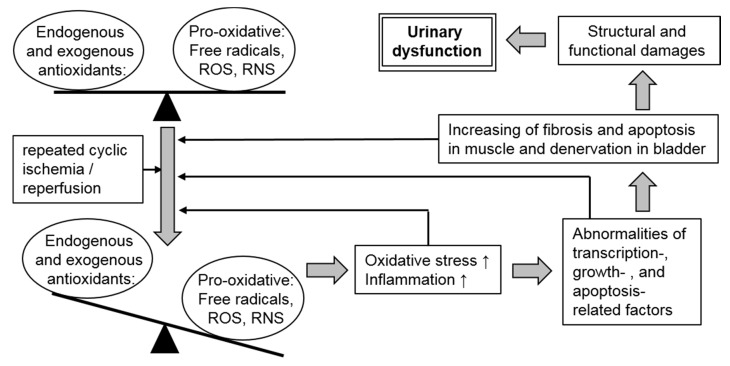
Oxidative stress is regulated by the balance between pro-oxidative and antioxidative factors. In partial bladder outlet obstruction (PBOO), repeated ischemia/reperfusion lead to excessive oxidative stress and increased inflammation due to an imbalance in these factors. Such pathological conditions stimulate abnormalities in transcription and cell-survival-related factors, which cause pathological changes in bladder tissues, such as denervation and increasing fibrosis and apoptosis in muscles. In turn, such oxidative stress-induced biological and histological changes in the bladder promote an imbalance in pro-oxidative and antioxidative factors. Finally, this vicious cycle that begins with cyclic ischemia/reperfusion in PBOO leads to further urinary dysfunction.

Prolonged and chronic cyclical ischemia/reperfusion produce ROS, and elevated ROS induce oxidative stress in the bladder and play important roles in bladder dysfunction via changes in cellular and molecular characteristics, including the density of blood vessels and nerves, and the induction of fibrosis [10,11,26]. In addition, ROS has been implicated in PBOO-induced bladder dysfunction because they damage detrusor mitochondria, consequently depressing energy production and impairing detrusor contractility [27]. Moreover, in a rat PBOO model, aging enhanced sensitivity of detrusor contraction to ROS-related oxidative damage [28]. Interestingly, reperfusion events mediated by ROS have shown greater damage than ischemic events alone in a rat model [29]. Based on this evidence, ROS is believed to be a major pathogenic factor for bladder dysfunction caused by PBOO. A schema of relationship between urinary dysfunction and oxidative stress in BOO is showed in Figure 1. 

## 3. Changes in Oxidative Biomarkers in Partial Bladder Outlet Obstruction

Numerous biomarkers are reported to reflect oxidative stress, and most of these oxidative stress markers indicate damage and oxidation of lipids, proteins, and DNA/RNA [30]. The most frequently used oxidative marker is 8-hydroxy-2-deoxyguanosine (8-OHdG), which is an oxidized product of DNA. In addition, malondialdehyde (MDA) is considered to be a useful oxidative stress marker that results from lipid peroxidation [31]. Isoprostanes (IsoPs) are classified as prostaglandin isomers, and they are major oxidative stress markers because they are end products of lipid peroxidation stimulated by free radicals [31,32]. In addition, F2-isoprostanes (F2-IsoPs) are chemically stable metabolic products produced by ROS that are detectable in all tissues and fluids [33,34,35]. F2-IsoP is considered by some researchers to be one of most useful markers for oxidative stress in vivo [36]. 

In PBOO, these markers are commonly used to evaluate the degree of oxidative stress. For examples, urinary levels of 8-OHdG were significantly higher relative to those of sham in a rat PBOO model [13]. Similar results regarding the relationship between urine 8-OHdG levels and PBOO have been reported elsewhere [25,37,38,39]. In addition, increased levels of 8-OHdG and MDA in blood samples from PBOO models compared with the control ones have been reported by several investigators [25,39,40,41]. In addition to oxidative stress markers in urine and blood, a decrease in mitochondrial DNA copy number and increase in 8-OHdG content were detected in the bladder tissues of a rabbit PBOO model [39]. MDA seems to be the most commonly used biomarker in blood. F2-IsoP has been measured in tissue samples only, and there was one report that F2-IsoP levels in bladder tissues of PBOO mice were significantly higher than those in the control [35]. Interestingly, elevation of tissue F2-IsoP levels was not related with aging [35]. In contrast, another study showed that F2-IsoP levels in bladder tissues of a PBOO mouse model were mildly increased immediately after bladder distention; however, these levels returned to normal after 24 h, and the levels in PBOO mice after three and five days of bladder distention were similar to those in the control [42]. There is no clear way to resolve the discrepancy between changes in F2-IsoP levels by PBOO in these two studies. However, the authors of the latter study suggested that oxidative stress events might still be occurring at three and five days of distension, and that chronic distension might stimulate cellular free radical detoxifying systems [42]. 

We summarized the changes in oxidative stress biomarkers in urine, blood, and tissue samples in Table 1. 

As mentioned above, almost all studies on 8-OHdG were performed with urine samples. Conversely, there is no data on other biomarkers in urine samples. On the contrary, F2-IsoP has been evaluated in tissue samples but not in urine or blood. Limitations regarding measurement techniques and concentrations of biological markers in each sample are beyond the scope of this review. However, systematic results regarding changes in multiple oxidative stress markers in each sample are important to a discussion of the etiology, pathological significance, and potential therapeutic targets in patients with PBOO. 

## 4. Antioxidants and Bladder Outlet Obstruction

Various antioxidant defense systems, which involve scavengers of free radicals that neutralize excessive ROS, protect against harmful activities caused by oxidative stress. Important factors in such antioxidant systems comprise various enzymes such as superoxide dismutase (SOD), catalase (CAT), and glutathione peroxidase (GSH) [31,49]. PBOO is reported to increase production of ROS, which leads to increased MDA and decreased SOD [50]. In addition, cooperation of these antioxidants is important to defend against oxidative stress under pathological conditions. For example, SOD converts reactive oxygen to hydrogen peroxide, which has a reactive radical capability, and CAT and GPX further break down hydrogen peroxide to water and oxygen [31]. Therefore, when the levels and activities of free radical scavengers are decreased in cells, oxidative stress is increased, and this increased oxidative stress can cause significant tissue damage. 

With regard to PBOO, changes in endogenous antioxidants have been shown in various animal experiments. The antioxidant activities of CAT, GSH, and SOD in PBOO models were significantly lower than those in the control in many of these experiments, but other reports have indicated that antioxidative activity in a rat PBOO model was similar or greater than that in the control (Table 2). We cannot explain this difference. Several reports have shown that plasma total antioxidant capacity (TAC), which is measured using a PAO kit (Nikken SEIL Co., Fukuroi, Japan) and reflects the cumulative effect of all antioxidants in a fluid sample, was decreased in a PBOO rabbit model [25,39]. Another study showed similar results regarding the relationship between TAC levels and PBOO using another commercial kit (Nanjing Jiancheng Bioengineering Institute, Nanjing, China) [47]. Based on this evidence, there is general agreement that antioxidative activity is decreased by PBOO, and that normalization of antioxidative status is a potentially valuable therapy for this disease.

## 5. Treatments Using Antioxidants for Bladder Outlet Obstruction

Transurethral resection is a standard treatment for patients with BOO and BPH. However, various complications such as blood transfusion, urge incontinence, and urinary tract infection are associated with this method and may occur during or after surgery [52]. Other conservative treatments, including treatment with α-blockers, phosphodiesterase type 5 inhibitors, and anti-cholinergic agents, are commonly used for patients with BOO exhibiting lower urinary tract symptoms (LUTS) [53]. Unfortunately, however, use of these agents often has side effects such as hypotension, dizziness, dry eye, and constipation. In addition, their effects are not sufficient to reduce symptoms in some patients. Therefore, information on new and additional treatment strategies for BOO is important. As mentioned above, antioxidants are potential therapeutic agents for LUTS caused by PBOO because induced ROS and free radicals due to cyclic ischemia/reperfusion have been implicated in the pathogenesis of bladder dysfunction [40,54]. Antioxidants comprise endogenous enzymes, metal-binding proteins, and exogenous dietary compounds [31]. For a discussion of therapeutic strategies using antioxidants for PBOO, information on the effects and safety of exogenous antioxidants and their dietary sources is needed. Vitamins, carotenoids, and polyphenols are well-known exogenous antioxidants [31]. In this section, we introduce treatment strategies based on dietary foods, fluids, and supplements with antioxidative effects. 

### 5.1. Eviprostat

Eviprostat, which is a phytotherapeutic agent composed of several plant extracts, has been used to treat urinary symptoms of benign prostatic hyperplasia (BPH) in Japan and Germany. A clinical study showed that this agent improved symptom scores and objective urinary condition-related parameters, including urinary flow rates [55]. In addition, the authors found that Eviprostat had anti-inflammatory effects when resected prostate specimens were investigated [55]. In cell-free systems and human neutrophils, Eviprostat has shown anti-inflammatory and antioxidant effects through a decrease in ROS levels [56]. There is also a report that this agent decreased urinary levels of 8-OHdG increased by BPH, and this anti-oxidative effect was confirmed in patients with LUTS associated with BPH [57]. Other investigators have shown that Eviprostat protects bladder function and pathological changes in the bladder wall, including hemorrhage, accumulation of leukocytes, and edema via suppression of oxidative stress induced by BOO in a rat model [37,58]. In addition, various cytokines are associated with the protective effects of Eviprostat [37]. Finally, antioxidant and anti-inflammatory activities of Eviprostat are responsible for beneficial effects observed in the treatment of BOO, including BPH. 

### 5.2. Alpha1-Adrenoreceptor Antagonists

In BPH patients, alpha (α)1-adrenoreceptor antagonists are commonly used worldwide. With regard to α1-adrenoreceptor antagonist and ischemia/reperfusion damage, naftopidil is reported to restore the decreased bladder blood flow of BOO to normal level in rats [59]. In addition, this study showed that more hypertrophic detrusor muscle and inflammatory cells were detected in the bladder tissues of BOO, whereas these pathological changes were suppressed with naftopidil treatment [59]. Regarding oxidative stress, this study also showed that although 8-OHdG levels in the BOO group were significantly higher than those in the control group, levels in the BOO + naftopidil group were significantly lower than those in the BOO group and similar to those in the control group [59]. Based on this evidence, there is the possibility that naftopidil restores bladder function via a reduction in oxidative stress and increase in bladder blood flow in BOO. Binding affinity for α1-adrenoreceptor subtypes are dependent on antagonist types. Naftopidil has a higher affinity for the α1_D_-adrenoreceptor than for α1_A_-adrenoreceptor subtype [60]. On the contrary, silodosin is an α1-adrenoreceptor antagonist with high binding affinity for the α1_A_-adrenoreceptor [61]. Regarding the antioxidative effects of silodosin, experiments with rats showed that silodosin significantly decreased MDA in bladders and that 8-OhdG levels in urine increased with chronic bladder ischemia “without” BOO [62]. Following this study, the same research group showed similar antioxidative effects of silodosin in a BOO rat model [62]. Finally, they concluded that silodosin improved urinary conditions, including voiding frequency and voided volume, via normalization of oxidative stress and recovery of bladder blood flow in BOO rats [62]. 

### 5.3. Melatonin

Melatonin is known to play important roles in various physiological functions, including the control of seasonal reproduction, the immune system, and the circadian rhythm [63]. In addition, melatonin is a potent antioxidant that suppresses oxidative stress caused by PBOO in animal models [44,45]. In short, tissue levels of MDA in a rabbit PBOO model were significantly higher than those in sham PBOO rabbits; however, these levels in a PBOO model were decreased to those of the sham by treatment with melatonin [45]. In addition, these researchers also found that decreased tissue levels of CAT, GSH, and SOD, which are antioxidants, in PBOO recovered to the levels in sham rabbits with melatonin treatment, whereas such significant effects were not detected following treatment with terazosin, which is an α1-adrenoreceptor antagonist [45]. However, interestingly, monotherapy with melatonin or terazosin did not significantly restore the contractility response of isolated bladder strips to KCl; however, rabbits treated with a combination of the two agents showed bladder contractility similar to that of the sham-operated ones [45]. On the contrary, some researchers have suggested that melatonin inhibits smooth muscle contractility [44]. Thus, melatonin may protect urinary function via multiple and complex mechanisms, and α1-adrenoreceptor antagonists are speculated to play significant roles in regulating oxidative stress. 

### 5.4. Alipoic Acid

Nitric oxide synthase (NOS) is closely associated with ROS/RNS function, and NOS-mediated ROS/RNS production plays crucial roles in various pathological conditions [21,50]. Endothelial NOS (eNOS), inducible NOS (iNOS), and neuronal NOS (nNOS) are distinct isoforms of NOS, and several investigators have examined the relationships between PBOO and NOS or iNOS [50,64,65]. For example, in a rabbit PBOO model, pre-medication with N(G)-nitro-l-arginine methyl ester (l-NAME), which is an inhibitor of NOS, can protect against oxidative damage [65]. Furthermore, inhibition of iNOS activity by knockout of iNOS and pharmacological methods significantly attenuated the increase in bladder size and the number of spontaneous bladder contractions in a rat PBOO model [64]. Thus, NOS, especially iNOS, is a potential therapeutic target for PBOO. Here, we introduce alipoic acid as an antioxidant because it inhibits iNOS activity. Alipoic acid, which is known as an ideal and unique antioxidant, is reported to protect bladder function against cyclic ischemic/reperfusion injury in a rat BOO model, and the inhibitory effects of alipoic acid against iNOS, collagen formation, lipid peroxidation, and TNF-α are associated with this protective mechanism [40]. Briefly, iNOS mRNA levels, the percentage of apoptotic cells measured by TdT-dUTP nick end labelling (TUNEL), and MDA levels in serum and tissues in the PBOO group were restored with alipoic acid treatment [40]. In addition, this study also showed that silymarin, which is related to oxidative stress, carcinogenesis, and apoptosis, also served as an antioxidant in the same model. However, in contrast to alipoic acid, silymarin showed no significant effect on type III collagen, apoptosis, or iNOS mRNA in bladders. Finally, the authors concluded that alipoic acid may be useful in protecting bladder tissues from BOO.

### 5.5. Vitamin E

Vitamin E, which is alpha-tocopherol, can reduce the frequency of bladder dysfunction in a BOO rabbit model, and, interestingly, a decrease in MDA level and increase in alpha-tocopherol concentration was detected in bladders with high-dose administration [66]. Based on this evidence, the authors concluded that at least part of the bladder dysfunction caused by PBOO is associated with free radical generation and resultant lipid peroxidation [66]. In addition, other investigators have shown that vitamin E protects against oxidative stress, including lipid peroxidation in rabbit bladder smooth muscle [67]. Unfortunately, the vitamin-E-mediated antioxidative effects in PBOO are not so clear. However, there is a report that, in a large cohort of elderly men given dietary antioxidants, including vitamin E, there was no significant effect of treatment on lower urinary tract symptoms [68]. 

### 5.6. Hydrogen Water

Hydrogen (H_2_) has been reported to be an antioxidant, and water with H_2_ (dissolved water, HW) has beneficial effects on ischemia/refusion injury in neural diseases, metabolic syndrome, and cardiovascular diseases [69,70,71]. Furthermore, H_2_ can selectively suppress harmful effects caused by strong oxidants; however, it does not change the physiological effects of ROS [69]. Based on this evidence, the antioxidative effects of H_2_ in urinary dysfunction was evaluated in a rat model of BOO [13], and a decreased micturition interval and volume in PBOO rats was observed with oral administration of H_2_ water. In addition, increased post-void residual urine in PBOO was significantly reduced with H_2_ water. Urine and bladder tissue levels of 8-OHdG and tissue levels of MDA in BOO rats given ordinary drinking water were significantly higher than those in the sham rats, and these increased levels were reduced with administration of H_2_ water [13]. Finally, the authors concluded that H_2_ water could protect bladder functions against BOO via suppression of oxidative stress. 

### 5.7. Omega-3 Fatty Acid

Omega-3 fatty acid is known as an essential fatty acid and is recognized as one of the most important structural components of cell membranes. In addition, omega-3 fatty acid has anti-inflammatory and antioxidative actions [72]. In a rat BOO model, bladder weight and fibrosis in PBOO rats orally administered omega-3 fatty acid for four weeks were significantly lower than those with BOO rats [43]. Furthermore, this study showed that omega-3 fatty acid treatment significantly modulated levels of bladder CAT, SOD, and MDA and serum SOD and GSH-Px that were affected by BOO [43]. The same study also showed that subcutaneous interferon α-2b administration partially decreased inflammation and oxidative stress induced by BOO. However, the synergic effects of these two agents on bladder structure and antioxidation are not clear. Many basic and clinical studies support the idea that omega-3 fatty acid has beneficial effects in various diseases, especially cardiovascular ones [73]. However, to our knowledge, there is just one report on the relationships between omega-3 fatty acid and oxidative stress, inflammation, and pathology in the bladder, and the authors of this study concluded that prospective randomized clinical trials with larger study populations are necessary [43]. We agree with their opinion and hope to perform these in the near future.

### 5.8. Coenzyme Q10, Alpha Lipoic Acid, and Their Combination

Coenzyme Q10 is a vitamin-like substance and a liquid-soluble cofactor detected in mitochondria. Coenzyme Q10 has antioxidant and anti-apoptotic activities in mitochondria and neurons [74]. Coenzyme Q10 significantly protected ischemia/reperfusion-damaged neurons against pathological changes and increased apoptosis of detrusor cells in a rabbit model [75]. Similar protective effects against bladder dysfunction and histological changes in the bladder wall following chronic bladder ischemia were confirmed in a rat model [76]. However, we should note that these reports showed beneficial effects against bladder dysfunction and pathological changes in the bladder after ischemia/reperfusion bladder injury, strictly speaking, rather than in PBOO. As mentioned above, ischemia/reperfusion bladder injury is a primary cause of bladder dysfunction and lower urinary tract symptoms in PBOO, but further animal experiments and clinical trials are needed to investigate the clinical and pathological efficacy of coenzyme Q10. 

Alpha lipoic acid (ALA) is an organic compound and an essential coenzyme in the oxidation of mitochondrial pyruvate and α-ketoglutarate [77]. There is general agreement that ALA acts as an ideal and universal antioxidant and as an ROS scavenger in the body, including in the bladder [78]. There is a report that ALA treatment protects bladder function via downregulation of stimulated oxidative stress and collagen formation caused by BOO in a rat model [40]. In addition, the same study showed that silymarin, which has anti-apoptotic and antioxidant effects, decreased oxidative stress in the urinary bladder [40]. On the contrary, Juan et al. paid special attention to the beneficial effects of combined therapy coenzyme Q10 and ALA, on bladder dysfunction caused by BOO [79]. They found that coenzyme Q10 plus ALA treatment significantly restored contractile responses and prevented detrusor smooth muscle hypertrophy in a rabbit BOO model, and speculated that the protective effects of these two strong antioxidants against free radical-induced bladder tissue damage are associated with their findings [79,80].

### 5.9. Sulforaphane

Sulforaphane is a naturally occurring isothiocyanate found in certain vegetables such as broccoli, cabbage, and cauliflowecauliflower. There is a report that sulforaphane treatment inhibits the increase in collagen fiber and apoptosis in bladder tissues caused by BOO and can improve bladder function in a rat model [41]. In addition, this study showed that such protective effects are mediated by the antioxidative effects of sulforaphane, because levels of the oxidative stress marker MDA in the bladder were increased and activities of CAT, GSH, and SOD in the bladder were decreased in a BOO model; however, these parameters returned to levels found in sham rats with sulforaphane treatment [41]. Furthermore, they found that changes in expression of apoptosis-related molecules such as Bcl-2 and Bax, percentage of apoptotic cells, and Nrf2 expression in the bladder were associated with the antioxidative effects of sulforaphane [41]. 

### 5.10. Xian-Jia-Tang

Xian-Jia-Tang, which is a traditional Chinese medicine, has been reported to suppress the increase in bladder volume weight and abnormal changes in urodynamic parameters in a rat model via inhibition of oxidative stress and potassium channels [47]. Briefly, MDA levels in detrusor muscles in a rat PBOO model were significantly higher than those in the normal control; however, increased MDA levels in detrusor muscles recovered to normal levels with Xian-Jia-Tang treatment [47]. Similarly, decreased levels of total antioxidative activity (T-AOC), which is endogenous antioxidant, in PBOO rats returned to normal levels with Xian-Jia-Tang treatment. Interestingly, they also found that such antioxidative effects of Xian-Jia-Tang were weakened by cesium chloride, which is a potassium channel inhibitor. Finally, they concluded that further investigations on active ingredients and these interactions are needed because Xian-Jia-Tang includes various agents. We support this opinion. However, there is scant literature in English on the pharmacological activities, clinical effects in a large study population, and adverse events of Xian-Jia-Tang. These limit further discussion on treatment strategies using this medicine.

### 5.11. Green Tea Polyphenol

Tea is one of most popular beverages in the world; however, green tea is mainly consumed in eastern countries such as China and Japan. Green tea contains various polyphenols, and many investigators have examined their pharmacological effects for the prevention and treatment of malignancies, cardiovascular diseases, and infectious diseases [81,82,83,84,85]. Epigallocatechin-3-gallate (EGCG) is a major component of green tea polyphenol, and it has many functions, including anti-inflammatory, anticancer, and antioxidative stress functions [81,83,84,85,86]. A previous report showed that EGCG reduced bladder injury and dysfunction caused by BOO via modulation of histologic changes, inflammation, and endoplasmic reticulum stress-related apoptosis in a rat model [87]. In addition, this study showed that cyclooxygenase-2, caspase-12, and CCAAT/-enhancer-binding protein homologous protein are regulated by EGCG [87]. There is also a report that intra-peritoneal injection of EGCG (5 mg/Kg) can protect urinary function, including compliance and micturition interval, in a rat BOO model [48]. In addition to such cystometric parameters, oxidative stress, measured by MDA, SOD, GSH, CAT, and total ROS activities, is mediated by EGCG administration, and the authors concluded that EGCG protects bladder function via regulation of oxidative stress [48]. Further, they found that EGCG is associated with changes in various inflammation- and apoptosis-related molecules such as Nrf2, caspase-3 and hemeoxygenase-1, and that such regulative mechanisms were useful in the protection of bladder function [48]. 

### 5.12. Tempol

Tempol, which is a free radical scavenger, was recently found to prevent BOO-induced thickening of collagen fibers and deposition of these fibers in the detrusor muscle layer via suppression of oxidative stress in a rat model [88]. In this model, the thickness and deposition of collagen fibers in the detrusor muscle layer in rats treated with tempol were significantly less than those in rats that were untreated, and the number of non-voiding contractions per voiding cycle decreased with tempol treatment. In addition, levels of the oxidative stress marker MDA in bladder tissues were significantly lower in tempol-treated rats than in untreated ones [88]. 

### 5.13. Edaravone

Edaravone is a substituted 2-pyrazolin-5-one class radical scavenging agent [89]. One study using a rat model showed that edaravone protected against ischemia/reperfusion injury in the urinary bladder, including morphological changes in smooth muscle and abnormalities in the contractile response [51]. In addition, they found increased levels of MDA in the bladder due to ischemia/reperfusion injury were reduced to control levels with edaravone treatment. Moreover, other investigators have reported that edaravone prevents rat bladder dysfunction caused by acute urinary retention and subsequent catheterization via suppression of oxidative stress [90].

A summary of changes in antioxidant and oxidative stress biomarker levels after treatment with these antioxidants is shown in Table 3.

## 6. Problems to be Solved and Future Directions

We agree that further detailed and systematic examinations of the relationships between degree, type, and duration of urinary dysfunction and each oxidative stress biomarker in various samples such as urine, blood, and bladder tissues are needed [80]. For example, in rats, a severe PBOO model with a 3Fr catheter (diameter 1 mm) showed increasing levels of MDA in bladder tissue compared to the control, whereas an intermediate PBOO model with a 4Fr catheter (1.3 mm) showed no significant difference [46]. In addition, as shown in Table 2, there are few biomarkers of oxidative stress in urine other than 8-OHdG. However, measurements of F2-IsoP in urine and blood samples are important for examining the pathological roles of oxidative stress in bladder dysfunction because F2-IsoP has only been measured in bladder tissues [35]. 

Moreover, we noticed that there is no appropriate and high-quality in vitro PBOO model for investigation of the detailed pathological roles of oxidative stress based on the balance between pro-oxidative and antioxidative factors. Furthermore, it is difficult to perform in vivo studies using bladder tissues of patients with PBOO because collection of bladder tissues is not necessary in standard methods of diagnosis and treatment. Because of this, animal experiments on rats and rabbits are performed. Many investigators believe that these animal models are suitable to discuss the pathological significance of oxidative stress and efficacy of antioxidants in PBOO. However, data obtained from human tissues of PBOO patients is crucial, especially in determining the efficacy and safety of antioxidant-based treatments. 

Comprehensive information is essential to discussing promising treatment strategies for patients with BOO. In other words, in addition to oxidative stress, various factors are associated with the etiology and development of bladder dysfunction caused by PBOO. For example, inflammatory cytokines, immune responses, nitric oxide synthases, and various growth factors also play important roles [46,50,91,92]. Furthermore, several transcriptional factors, including nuclear erythroid 2-like 2 and nuclear kappa B play important roles in urinary dysfunction via the cellular response to oxidative stress [46,48]. 

Based on previous reports, we think that a single therapy using an antioxidant agent may not be sufficient to reduce symptoms and obtain objective data in patients with PBOO. However, there is the possibility that therapies with a combination of antioxidant(s) and other agent(s) may be useful for these patients. Therefore, we emphasize the importance of further clinical trials with large study populations. 

## 7. Conclusions

In this review, we summarized reports on oxidative stress and antioxidants in PBOO, mainly based on the results of animal experiments. These reports strongly support the idea that oxidative stress plays an important role in the pathogenesis of urinary dysfunction in PBOO. Various drugs and supplements with anti-oxidant activities have been reported to improve subjective and objective measures via suppression of oxidative stress in PBOO models. Unfortunately, however, their effects are unconfirmed in almost all cases. Although there are many reports on biomarkers of oxidative stress in animal models, the detailed pathological significance of such biomarkers is not fully understood. Based on this review, we would like to emphasize that more comprehensive information from a wide range of studies is needed to develop treatment strategies for patients with PBOO. 

## Figures and Tables

**Table 1:** Changes of oxidative stress biomarkers in bladder outlet obstruction.

Biomarkers	Species	Changes	Sample	Author/Year/References
DNA base oxidation				
8-OHdG	Rats	Increased	Urine	Oka/2009/[37]
	Rabbit	Increased	Urine	Matsumoto/2010/[38]
	Human	Increased	Urine	Matsumoto/2010/[38]
	Rabbit	Increased	Urine	Lin/2011/[25]
	Rabbit	Increased	Urine	Lin/2012/[39]
	Rats	Increased	Urine	Miyazaki/2016/[13]
	Rabbit	Increased	Blood	Lin/2012/[39]
	Rats	Increased	Tissue	Miyazaki/2016/[13]
Lipid peroxidation				
MDA	Rabbit	Increased	Blood	Lin/2011/[25]
	Rabbit	Increased	Blood	Lin/2012/[39]
	Rats	Increased	Blood	Yildirim/2013/[40]
	Rats	No change	Blood	Firat/2016/[43]
	Rats	Increased	Blood	Liu/2016/[41]
	Rat	Increased	Tissue	Sener/2003/[44]
	Rabbit	Increased	Tissue	Onur/2008/[45]
	Rats	Increased	Tissue	Oka/2009/[37]
	Rats	Increased	Tissue	Yuan/2011/[44]
	Rats	Increased	Tissue	Yildirim/2013/[40]
	Rats	No change	Tissue	Firat/2016/[43]
	Rats	Increased	Tissue	Miyazaki/2016/[13]
	Rats	Increased	Tissue	Liu/2016/[41]
	Rats	Increased	Tissue	Sezginer/2017/[46]
	Rats	Increased	Tissue	Sun/2017/[47]
	Rats	Increased	Tissue	Gu/2018/[48]
F2-IsoP	Mice	No change	Tissue	Stephany/2013/[42]
	Mice	Increased	Tissue	Clayton/2014/[35]

8-OHdG, 8-hydroxy-2-deoxyguanosine; MDA, malondialdehyde; F2-IsoP, F2-isoprostane.

**Table 2:** Changes of endogenous antioxidants in bladder outlet obstruction.

Biomarkers	Species	Changes	Sample	Author/Year/References
CAT	Rabbit	Decreased	Tissue	Guven /2007/[51]
	Rabbit	Decreased	Tissue	Onur/2008/[45]
	Rats	No change	Tissue	Firat/2016/[43]
	Rats	Decreased	Tissue	Liu/2016/[41]
	Rats	Decreased	Tissue	Gu/2018/[48]
GSH	Rabbit	Decreased	Blood	Lin/2012/[39]
	Rats	Increased	Blood	Firat/2016/[43]
	Rabbit	Decreased	Tissue	Onur/2008/[45]
	Rats	Decreased	Tissue	Sener/2003/[44]
	Rats	No change	Tissue	Firat/2016/[43]
	Rats	Decreased	Tissue	Liu/2016/[41]
	Rats	Decreased	Tissue	Gu/2018/[48]
SOD	Rats	Increased	Blood	Firat/2016/[43]
	Rabbit	Decreased	Tissue	Guven/2007/[51]
	Rabbit	Decreased	Tissue	Onur/2008/[45]
	Rats	Decreased	Tissue	Yuan/2011/[50]
	Rats	Increased	Tissue	Firat/2016/[43]
	Rats	Decreased	Tissue	Liu/2016/[41]
	Rats	Increased	Tissue	Sun/2017/[47]
	Rats	Decreased	Tissue	Gu/2018/[48]
TAC	Rabbit	Decreased	Blood	Lin/2011/[25]
	Rabbit	Decreased	Blood	Lin/2012/[39]
	Rats	Decreased	Tissue	Sun/2017/[47]

CAT, catalase; GSH, glutathione peroxidase; SOD, superoxide dismutase; TAC, total antioxidant capacity.

**Table 3:** Changes in antioxidant and oxidative stress biomarker levels after antioxidant treatment.

Antioxidant	Changes in Antioxidant and Oxidative Stress Marker Levels	Reference
Eviprostat	Decreases urinary levels of 8-OHdG	[57]
Naftopidil	Suppresses urinary level of 8-OHdG	[59]
Silodosin	Decreases tissue levels of MDA and urine levels of 8-OHdG	[62]
Melatonin	Decreases tissue levels of MDA and recovers tissue levels of CAT, GSH, and SOD	[45]
Alipoic acid	Restores serum and tissues levels of MDA	[40]
Vitamin E	Decreases tissue levels of MDA	[66]
Hydrogen water	Reduces urine and bladder tissue levels of 8-OHdG and tissue levels of MDA	[13]
Omega-3 fatty acid	Increases tissue levels of CAT, SOD, and MDA and serum levels of SOD and GSH	[43]
Coenzyme Q10	Decreases tissue and serum levels of MDA	[76]
Sulforaphane	Decreases tissue levels of MDA and increases CAT, GSH, and SOD activities in the bladder	[41]
Xian-Jia-Tang	Recovers MDA levels in detrusor muscles to normal levels and increases tissue levels of T-AOC	[47]
Green tea polyphenol	Decreases tissues levels of MDA and increases SOD, GSH, and CAT	[48]
Tempol	Decreases tissue levels of MDA	[88]
Edaravone	Reduces tissue levels of MDA to control levels	[51]

8-OHdG, 8-hydroxy-2-deoxyguanosine; MDA, malondialdehyde; CAT, catalase; GSH, glutathione peroxidase; SOD, superoxide dismutase; TAC, total antioxidant capacity; T-AOC, total antioxidative activity.
